# The association of left ventricular fraction shortening with cardiovascular events in peritoneal dialysis patients

**DOI:** 10.1080/0886022X.2023.2261786

**Published:** 2023-10-01

**Authors:** Lu Dai, Yuqi Yang, Lu Liu, Changzhu Long, Jingjing Da, Shuang Chen, Jianqiu Zhao, Yan Shen, Chengchong Huang, Yan Zha, Jing Yuan

**Affiliations:** a Department of Nephrology, Guizhou Provincial People’s Hospital, Guiyang, China; b Key Laboratory of Diagnosis and Treatment of Pulmonary Immuned-related Diseases, NHC, Guiyang, China

**Keywords:** Left ventricular fraction shortening, cardiovascular events, peritoneal dialysis, echocardiography

## Abstract

**Background:**

Peritoneal dialysis (PD) patients have a high incidence of cardiovascular events (CVEs). Left ventricular fraction shortening (LVFS), one of the echocardiographic parameters, is an independent risk factor for mortality in previous studies. The aim of this study was to evaluate associations between LVFS and CVEs in PD patients.

**Methods:**

This was a single-center observational cohort study. Seven hundred and eighty-four PD patients were enrolled from 1 January 2012 to 1 June 2021 and followed until 1 June 2022. The primary outcome was the incidence of CVEs. PD patients were categorized into three groups according to the tertiles of LVFS levels (tertile 1-tertile 3). Kaplan-Meier method, Cox proportional hazard models and competing risk regression models were used for survival analysis. The areas under the curve (AUC) of receiver-operating characteristic analysis was used to determine the predictive values of LVFS for CVEs. A preplanned subgroup analysis was assessed according to age, gender, and the presence of hypertension and dyslipidemia, etc.

**Results:**

During a median follow-up period of 42.3 months (interquartile range 24.0–79.0 months), 259 CVEs occurred. Compared to the other two groups respectively, patients in tertile 3 group had the lowest incidence of CVEs (24.5% vs 31.6% vs 43.0%, respectively, *p* < 0.05). After multiple adjustments, the tertile 3 group was associated with the 45.1% decrease in the CVEs hazard compared to that of the tertile1 group (SHR = 0.549, 95%CI: 0.395–0.762, *p* < 0.001). Subgroup analysis demonstrated that tertile 1 group as the reference, the association between LVFS and CVEs in tertile 3 group was robust among female patients (HR = 0.506, 95%CI: 0.309–0.829, *p* = 0.007), aged < 45 years (HR = 0.496, 95%CI: 0.331–0.744, *p* = 0.001), history of hypertension (HR = 0.586, 95%CI: 0.349–0.872, *p* = 0.008) and combined with dyslipidemia (HR = 0.464, 95%CI: 0.269–0.799, *p* = 0.006).

**Conclusions:**

This study suggests that LVFS is independently associated with the increased risk of CVEs in PD patients, especially those with aged < 45 years, female, with hypertension and dyslipidemia.

## Introduction

Cardiovascular disease (CVD) is the major cause of mortality in patients undergoing peritoneal dialysis (PD), which comprises about 40%–60% of mortality [1]. Although the development of effective dialysis and medication treatment for dialysis patients have been improved, cardiovascular events (CVEs) related mortality is still predicted to be further increased by 2030 [2]. Conventional risk factors, such as age, smoking, obesity, do not completely explain the excessive morbidity and cardiovascular mortality in this population [3]. The important biochemical and physiological changes in PD patients such as the uremic toxicity, protein-energy wasting syndrome, inflammation, ultrafiltration failure and overhydration also attributed to the pathogenesis of CVEs [4]. Therefore, it is critical to develop novel risk factors of CVEs in PD patients, in order to allow early and aggressive interventions to attenuate the disease progression and improve prognosis.

Echocardiography is a simple, safe, and low-cost noninvasive diagnostic technology, and the echocardiographic parameters have been widely used to predict CVEs [5–7]. Left ventricular fraction shortening (LVFS), one of the echocardiography parameters, plays an important role in assessing the impact of chronic organic mitral regurgitation, ventricular remodeling, myocardial infarction related cardiac dysfunction, overload-induced cardiac hypertrophy, and survival [8–10]. Previous studies show that echocardiographic parameters play an indispensable role in evaluating poor outcomes in chronic kidney disease (CKD) [11]. In addition, to our best knowledge, few studies have investigated the association between LVFS and clinical outcomes in PD patients. In this study, we aimed to investigate the associations between LVFS and CVEs in PD patients.

## Materials and methods

### Study patients and design

In this single-center observational cohort study, the patients commencing PD patients as the first kidney replacement therapy in the Department of Nephrology of Guizhou People’s Hospital from 1 January 2012 to 1 June 2021 were recruited. The inclusion criteria were as follows [1]: over 18 years old [2]; regular PD for more than 3 months. Exclusion criteria are as follows [1]: kidney transplantation or received hemodialysis prior to PD [2]; missing LVFS values.

**Figure 1.**
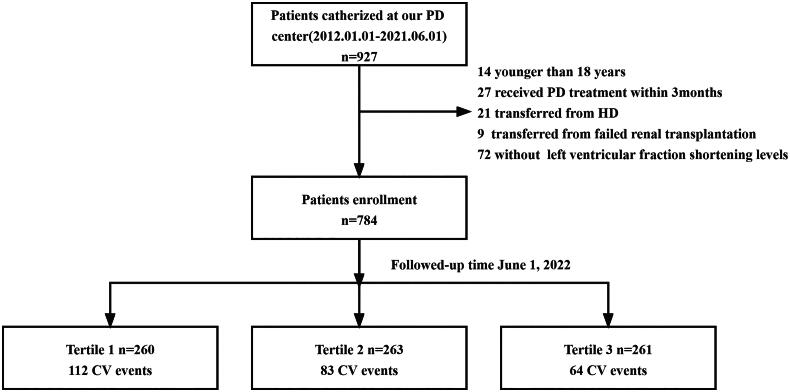
Flow chart of the study, PD: peritoneal; CV: cardiovascular.

The flow chart is shown in Figure 1. This study was conducted according to the ethical requirements of the Human Research Ethics Committee of Guizhou Provincial People’s Hospital ([2020] 208). All participants provided the written informed consent and all research procedures were conducted in accordance with relevant guidelines and regulations.

### Data collection

Baseline characteristics at the initiation of PD therapy were obtained from medical records, including gender, age, dialysis vintages, causes of end stage kidney disease (ESKD), history of hypertension and diabetes. Laboratory parameters were collected within 3 months after the initiation of PD. Including white blood cell, hemoglobin, platelet, albumin, triglyceride, total cholesterol, low-density lipoprotein cholesterol (LDL-C), high-density lipoprotein cholesterol (HDL-C), creatinine, uric acid, estimated glomerular filtration rate (eGFR), high-sensitivity C-reactive protein (Hs-CRP), calcium, phosphorus, parathyroid hormone, alkaline phosphatase, body mass index (BMI) was calculated as weight/height^2^ (kg/m^2^).

### Exposed events

Echocardiograms were performed before the initiation of PD treatment and after emptying the peritoneal cavity, which performed by two experienced, unified trained and qualified cardiologists. The patients were placed in a left decubitus position, and standardized two-dimensional and guided M-mode echocardiographic imaging was performed, left ventricular end diastolic dimension (Lvdd), left ventricular end-systolic diameter (Lvsd), interventricular septum thickness (IVS), posterior wall thickness of left ventricle, left atrial diameter (LAD), inner diameter of right atrium (RA) and ejection fraction (EF) were measured, LVFS = [(Lvdd - Lvsd)/(Lvdd)] × 100%.

### Outcome events

The CVEs endpoint was defined as having one or more of the following CVDs after the initiation of PD therapy: angina pectoris, myocardial infarction, heart failure, angioplasty, coronary artery bypass graft or stroke, interesting admission for fluid overload and pulmonary edema [12]. All follow-up and event evaluations were conducted by the pre-defined evaluation committee, which consisted of two specially trained, qualified, and clinically experienced internal medicine experts in charge. PD patients were followed up every six months, the patients were followed up until the incidence of CVEs, cessation of PD, kidney transplantation, transfer to other centers, all-cause death, or the end of follow-up on 1 June 2022, and patients who did not receive follow-up were followed up by telephone.

### Statistical analysis

The study population was divided into three groups based on the tertiles of LVFS levels. Continuous variables were expressed by mean ± standard deviation if normally distributed or median (25^th^–75^th^ percentile) if not normally distributed and categorical variables by frequencies and percentages. Differences among the LVFS groups were compared using the Kruskal-Wallis tests for continuous variables and the Chi-squared test for categorical variables. The Kaplan-Meier method was used to plot survival curves for cardiovascular event-free survival. The differences were assessed using the log-rank test. The time to CVEs were examined by Cox proportional hazards. Competing risk analysis and kidney transplantation was considered as the competing event was used to a sensitive analysis. The covariates for Cox proportional hazards models and competing risk analysis were the same. A univariate and multivariate analysis model analysis model was used to investigate the relationship between independent variable with outcomes, variables with *p* < 0.05 in univariate analysis and those associated with LVFS were included in multivariate regression analysis. Model 1 adjusted for age, gender, body mass index, hypertension, diabetes mellitus; Model 2: adjusted for dialysis vintage, total cholesterol, low-density lipoprotein cholesterol, albumin based on Model 1; Model 3: adjusted for C-reaction protein, calcium, phosphorus, use of antihypertensive drugs and cholecalciferol drugs based Model 2. The results were expressed as risk ratio (HR) and sub-distribution hazard ratios (SHR) with 95% confidence interval (95%CI). The LVFS values with the first triplicate were selected as the reference. Receiver operating characteristic curve (ROC) was used to analyze the predictive value of LVFS for CVEs in PD patients. Subgroup analyses stratified by age, gender, hypertension, BMI, hypoalbuminemia, diabetes and dyslipidemia were performed. Restricted cubic splines(RCS) were used to model and visualize the relation of LVFS with CVEs in PD patients. All data input and statistical analysis were conducted using SPSS version 26.0 (IBM Corp, Armonk, NY, USA), survival analysis, competing risk analysis, subgroup analysis and RCS were performed with the use of R, version 4.2.3 (http://www.R-project.org/). A two-tailed *p*-value < 0.05 was considered to indicate a statistically significant difference.

## Results

### Study population

A total of 927 incident PD patients between 1 January 2012 to 1 June 2021 in this study. Of these, 14 patients were younger than 18 years, 27 had received PD treatment for less than 3 months, 9 had transferred from failed kidney transplantation, 72 had missing data on LVFS levels. Finally, a total of 784 patients were enrolled in this study (Figure 1).

There were 444 males (56.6%), with a mean age of 42.3 ± 14.5 years old. The most common cause of kidney failure were glomerulonephritis (65.8%), followed by diabetic kidney disease (12.2%), hypertensive kidney disease (11.4%), and other reasons in (10.6%). They were divided into three groups according to the LVFS levels: tertile1 (≤ 31.0, *n* = 260); levels: tertile 2 (31.0–35.0, *n* = 263); levels: tertile 3 (>35.0, *n* = 261). Table 1 summarizes the demographic characteristics and laboratorial parameters among three LVFS groups. Compared with the other two groups, the patients in tertile1 had a higher prevalence of hypertension, levels of left ventricular diameter, lower levels of mean corpuscular volume, serum calcium, posterior wall thickness of left ventricle, and ejection fraction (*p* < 0.05 for each). There were no significant differences among groups in gender, diabetic kidney disease, BMI, hematocrit, albumin, creatinine, estimated glomerular filtration rate, phosphorus, parathyroid hormone, alkaline phosphatase, interventricular septum thickness (*p* > 0.05).

**Table 1.** Baseline characteristics of PD patients stratified by the LVFS levels.

Characteristics	Total (*n* = 784)	LVFS			
		Tertile 1(≤31.0) (*n* = 260)	Tertile 2(31.0–35.0) (*n* = 263)	Tertile 3(>35.0) (*n* = 261)	*P*-value
Men (n[%])	444 (56.6%)	144 (55.3%)	151 (57.4%)	149 (57.0%)	0.881
Age (years)	42.3 ± 14.5	40.0 ± 14.4	43.0 ± 14.1	43.0 ± 14.7	0.024
dialysis vintage (months)	42.0 (24.0,79.0)	38.4 (23.0, 76.0)	42.5 (24.0, 80.0)	49.0 (24.4, 82.0)	0.159
Primary cause of kidney failure					
Diabetic kidney disease (*n*[%])	96 (12.2%)	34 (13%)	26 (10%)	36 (13.7%)	0.348
Glomerulonephritis (*n*[%])	516 (65.8%)	187 (71.9%)	165 (62.7%)	164 (62.8%)	0.040
Hypertensive kidney disease (*n*[%])	89 (11.4%)	20 (7.7%)	39 (14.8%)	30 (11.4%)	0.036
Others (*n*[%])	83 (10.6%)	19 (7.3%)	33 (12.5%)	31 (11.8%)	0.106
Comorbidity					
Cardiovascular events (*n*[%])	259 (33%)	112 (43.0%)	83 (31.6%)	64 (24.5%)	< 0.001
Hypertension (*n*[%])	425 (54.2%)	163 (62.7%)	134 (50.9%)	128 (49.0%)	0.003
Diabetes (*n*[%])	157 (20.0%)	61 (23.4%)	43 (16.3%)	53 (20.3%)	0.450
Ultrafiltration volume	300 (100,500)	250 (125,500)	300 (150,600)	300 (150,500)	0.732
Body mass index (kg/m^2^)	22.5 (21.4,23.6)	22.4 (21.3,23.6)	22.5 (21.5,23.5)	22.6 (21.5,23.8)	0.105
White blood cell (×10^9^/L)	6.60 (5.36,7.79)	6.31 (5.15,7.43)	6.61 (5.45,7.76)	6.80 (5.58,8.20)	0.013
Hemoglobin (g/L)	99.8 ± 20.8	98.3 ± 19.9	99.3 ± 20.7	102.1 ± 21.9	0.040
Mean corpuscular volume (%)	88.5(84.7,92.1)	87.9(84.1,90.5)	88.6(84.7,92.1)	88.7(85.2,92.5)	0.034
Albumin (g/L)	34.6 ± 6.1	34.5 ± 6.1	34.8 ± 6.3	34.9 ± 6.2	0.243
LDL-C (mmol/L)	2.70 (2.37, 3.09)	2.71 (2.44, 3.14)	2.69 (2.29, 3.03)	2.73 (2.41, 3.09)	0.309
HDL-C (mmol/L)	1.16 (1.03, 1.32)	1.17 (1.05, 1.31)	1.13 (1.01, 1.31)	1.18 (1.05, 1.33)	0.133
Creatinine (µmol/L)	764.1 (611.2, 1000.5)	762.1 (611.8, 995.6)	757.2 (592.3, 966.5)	778.0 (621.5, 1023.0)	0.569
Uric acid (µmol/L)	403 (344, 465)	405 (351, 464)	414 (357, 468)	395 (339, 458)	0.229
eGFR (ml/min per 1.73 m2)	7.95 (6.34, 9.76)	7.9 (6.2, 9.7)	8.0 (6.3, 9.8)	7.5 (5.6, 9.5)	0.250
Hs-CRP (mg/L)	5.83 (2.03, 13.3)	4.6 (2.1, 11.9)	6.1 (2.3, 14.8)	6.5 (2.1, 14.3)	0.217
Calcium (mmol/L)	2.17 ± 0.24	2.15 ± 0.21	2.16 ± 0.26	2.20 ± 0.24	0.003
Phosphorus (mmol/L)	1.41 (1.19, 1.68)	1.45 (1.25, 1.72)	1.40 (1.18, 1.70)	1.37 (1.17, 1.68)	0.111
Parathyroid hormone (ng/mL)	315 (225, 451)	340 (241, 486)	305 (227, 449)	308 (218, 457)	0.082
Alkaline phosphatase (U/L)	81 (69, 97)	81 (70, 99)	79 (67, 95)	84 (71, 102)	0.108
IVS (mm)	9.3 (8.1, 10.65)	9.2 (8.0, 10.6)	9.3 (8.3, 10.4)	9.1 (8.0, 10.8)	0.648
LVPW (mm)	9.7 (8.6, 10.7)	9.9 (8.9, 11.0)	9.5 (8.2, 10.5)	9.3 (8.4, 10.4)	0.020
LAD (mm)	31.1 (27.0, 36.0)	34.7 (29.5, 37.3)	30.3 (26.9, 35.4)	29.0 (26.0, 33.4)	< 0.001
LVD (mm)	49.0 (45.4, 53.0)	52.2 (48.7, 57.0)	48.0 (45.0, 51.2)	47.7 (43.0, 50.0)	< 0.001
RA (mm)	40 (37, 43)	40 (37, 44)	40 (36, 43)	39 (36, 42)	0.002
EF (%)	61 (56, 65)	53 (46, 56)	61 (59, 63)	68 (65, 70)	< 0.001
LVFS (%)	33 (29, 36)	28 (24, 29)	33 (32, 34)	38 (36, 40)	< 0.001

Note: *p* < 0.05 was considered statistically significant. Values were expressed as mean ± standard deviation, median (25^th^–75th percentile), or frequency (percentage) as appropriate. Abbreviations: PD: peritoneal dialysis; LVFS, left ventricular fraction shortening; LDL-C: low-density lipoprotein cholesterol; HDL- C: high-density lipoprotein cholesterol; eGFR: estimated glomerular filtration rate; Hs-CRP: high-sensitivity C-reactive protein IVS: interventricular septum thickness; LVPW: posterior wall thickness of left ventricle; LAD: left atrial diameter; LVD: left ventricular diameter; RA: inner diameter of right atrium; EF: ejection fraction.

### Association of LVFS with the cardiovascular events in PD patients

During the median follow-up period of 42.3 months (interquartile range = 24.0–79.0 months), 442 (56.3%) patients continued PD treatment in our center, 54 (6.8%) underwent kidney transplantation, 143 (18.2%) transferred to hemodialysis treatment, 46 (5.8%) were lost to follow-up, 99 (12.6%) occurred all-cause mortality and 259 (33.0%) occurred CVEs (Table 2).

**Table 2.** Clinical outcomes of PD patients stratified by the LVFS.

Variables	Total (*n* = 784)	Left ventricular fraction shortening			
		Tertile 1 (≤ 31.0)	Tertile 2 (31.0–35.0)	Tertile 3 (>35.0)	*P*-value
		(*n* = 260)	(*n* = 263)	(*n* = 261)	
Follow-up (months)	42.3 (24.0, 79.0)	38.0 (22.9, 76.0)	42.0 (23.9, 79.0)	48.0 (25.0, 82.0)	0.002
Cardiovascular event (*n*[%])	259 (33.0%)	112 (43.0%)	83 (31.6%)	64 (24.5%)	< 0.001
Death (*n*[%])	99 (12.6%)	41 (15.7%)	36 (13.6%)	22 (8.4%)	0.151
Kidney transplantation (*n*[%])	54 (6.8%)	21 (8.1%)	20 (7.6%)	13 (4.9%)	0.438
Other centers (*n*[%])	47 (5.9%)	17 (6.5%)	18 (6.8%)	10 (3.8%)	0.725
transferred to HD (*n*[%])	143 (18.2%)	49 (18.8%)	48 (18.2%)	46 (17.6%)	0.254
Lost to follow-up (*n*[%])	46 (5.8%)	19 (7.3%)	15 (5.7%)	12 (4.5)	0.733

**Figure 2.**
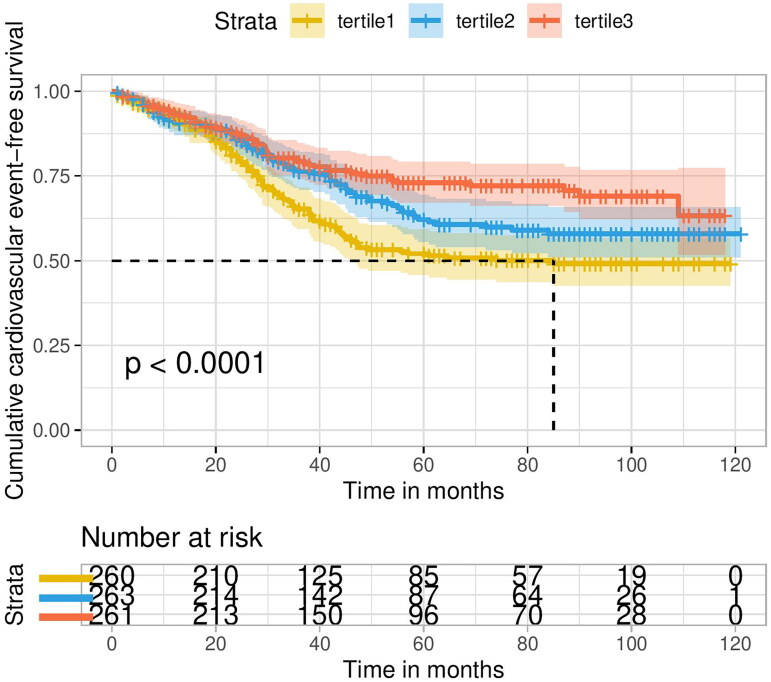
Survival curves of patients stratified according to left ventricular fraction shortening.

Survival curves of patients stratified according to different LVFS levels are shown in Figure 2. The participants in the tertile1 group had a significantly higher incidence compared to those in the other two groups of LVFS (log-rank = 11.810, *p* < 0.0001). The similar trend of CVEs in PD patients was observed in Cox proportional hazards models (Table 3). In the univariate model, the risk of CVE in group tertile 2 and tertile 3 were 28.9% (HR = 0.711, 95%CI: 0.535–0.944, *p* = 0.018) and 47.9% (HR = 0.521, 95%CI: 0.383-0.708, *p* < 0.001). After multiple adjustment, the risk of CVE in group tertile 2 and tertile 3 were 32.2% (HR = 0.678, 95%CI: 0.503–0.915, *p* = 0.011) and 48.6% (HR = 0.514, 95%CI: 0.374–0.706, *p* < 0.001).

**Table 3.** Cox proportional hazards models of baseline LVFS, EF and cardiovascular events.

Variables	Unadjusted Model		Model 1		Model 2		Model 3	
	HR (95%CI*)*	*P*-value	HR (95%CI)	*P*-value	HR (95%CI)	*P*-value	HR (95%CI)	*P*-value
Continuous variable								
LVFS	0.953 (0.934–0.973)	<0.001	0.948 (0.929-0.969)	<0.001	0.949 (0.930-0.969)	<0.001	0.950 (0.930-0.970)	<0.001
Categorical variable								
LVFS tertile 1	Reference	–	Reference	–	Reference	–	Reference	–
LVFS tertile 2	0.711 (0.535–0.944)	0.018	0.662 (0.494–0.888)	0.014	0.665 (0.495–0.894)	0.007	0.678 (0.503–915)	0.011
LVFS tertile 3	0.521 (0.383–0.708)	<0.001	0.493 (0.360–0.676)	<0.001	0.497 (0.362–0.691)	<0.001	0.514 (0.374–0.706)	<0.001
Continuous variable								
EF	0.970 (0.957-0.983)	<0.001	0.968 (0.955-0.981)	<0.001	0.968 (0.954-0.982)	<0.001	0.969 (0.955-0.981)	<0.001
Categorical variable								
EF tertile 1	Reference	–	Reference	–	Reference	–	Reference	–
EF tertile 2	0.732 (0.549–0.975)	0.033	0.708 (0.526–0.953)	0.043	0.712 (0.529–0.959)	0.025	0.719 (0.531–0.974)	0.033
EF tertile 3	0.601 (0.442–0.816)	<0.001	0.575 (0.420–0.787)	0.001	0.578 (0.422–0.791)	0.001	0.594 (0.433–0.817)	0.001

Model 1 is adjusted for age, gender, body mass index, hypertension, diabetes mellitus.

Model 2 is adjusted for age, gender, body mass index, hypertension, diabetes mellitus, dialysis vintage, total cholesterol, low-density lipoprotein cholesterol, albumin;.

Model 3 is adjusted for age, gender, body mass index, hypertension, diabetes mellitus, dialysis vintage, total cholesterol, low-density lipoprotein cholesterol, albumin, C-reaction protein, calcium, phosphorus, use of antihypertensive drugs and cholecalciferol drugs.

Abbreviations: CI: confidence interval; HR: hazard ratio; LVFS: left ventricular fraction shortening; EF:ejection fraction, *p* < 0.05 was considered statistically significant.

However, in one sensitivity analysis, we further explored the relationship between reduced LVFS and CVEs in PD patients using a competitive risk regression models with kidney transplantation as the competing event. This remained the case, in the competing risk analysis with high LVFS levels (tertile 3) associated with a lower risk compared to tertile 1 (SHR = 0.549, 95% CI: 0.395–0.762, *p* < 0.001) after multiple adjustment (Table 4). In addition, Cox proportional hazards models of baseline EF, LAD, left ventricular diameter (LVD), interventricular septum thickness (IVS), posterior wall thickness of left ventricle (LVPW) and CVEs are given in Supplemental Table 2.

**Table 4.** Sensitive analysis :Multivariate competing risk regression^a^ analysis for cardiovascular events^b^.

LVFS	cardiovascular events	
	SHR(95%CI)	*p* value
Tertile 1 (LVFS ≤ 31.0)	Reference	–
Tertile 2 (LVFS:31.0-35.0)	0.716 (0.534–0.595)	0.025
Tertile 3 (LVFS > 35.0)	0.549 (0.395–0.762)	<0.001

^a^Adjusted for age, gender, body mass index, hypertension, diabetes mellitus, dialysis vintage, total cholesterol, low-density lipoprotein cholesterol, albumin, C-reaction protein, calcium, phosphorus, use of antihypertensive drugs and cholecalciferol drugs.

^b^Kidney transplantation as a competing event; SHR: sub-distribution hazards ratio. *p* < 0.05 was considered statistically significant.

Subgroup analysis were conducted by age, gender, hypertension (Figure 3a), BMI, hypoalbuminemia, diabetes and dyslipidemia (Figure 3b). The results, compared to the tertile 1 group respectively, the association of the LVFS with cardiovascular events in tertile 3 group was robust among female patients (HR = 0.506, 95%CI: 0.309–0.829, *p* = 0.007), aged < 45 years (HR = 0.496, 95%CI: 0.331–0.744, *p* = 0.001), history of hypertension (HR = 0.586, 95%CI: 0.349–0.872, *p* = 0.007), BMI < 24.0 kg/m2 (HR = 0.460, 95%CI: 0.323–0.654, *p* < 0.001), without hypoalbuminemia (HR = 0.428, 95%CI: 0.331–0.661, *p* < 0.001), history of without diabetes (HR = 0.448, 95%CI: 0.308–0.650, *p* < 0.001) and with dyslipidemia (HR = 0.464, 95%CI: 0.269–0.799, *p* = 0.006). Moreover, the association of the MHR with CVEs was significantly modified by hypertension (*p* for interaction = 0.015).

Figure 3.**a**: Subgroup analyses of the association between LVFS and cardiovascular events among patients on peritoneal dialysis **3.b**: Subgroup analyses of the association between LVFS and cardiovascular events among patients on peritoneal dialysis.

**Figure:**
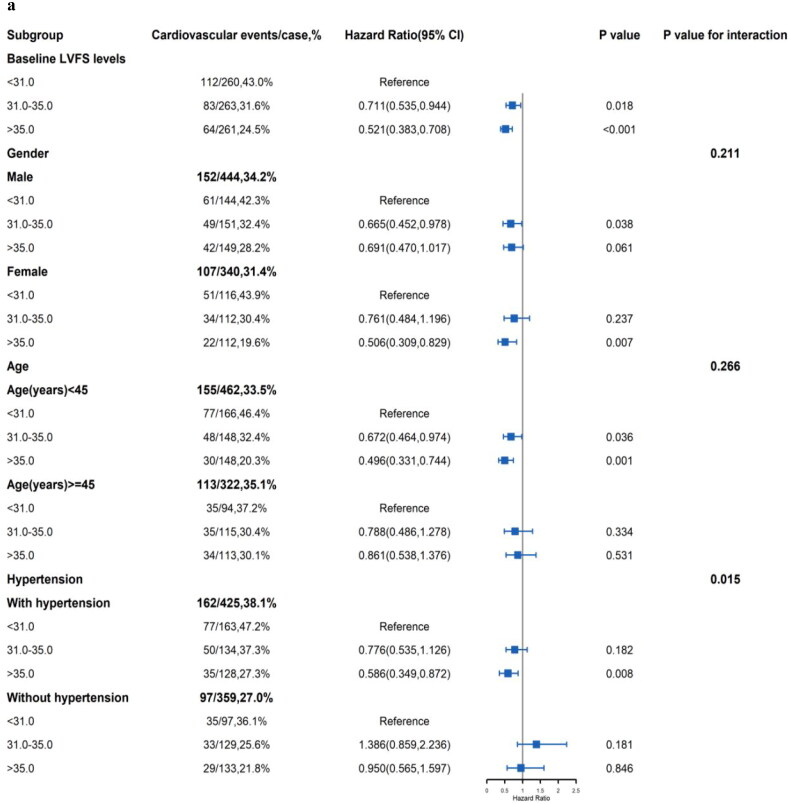

**Figure:**
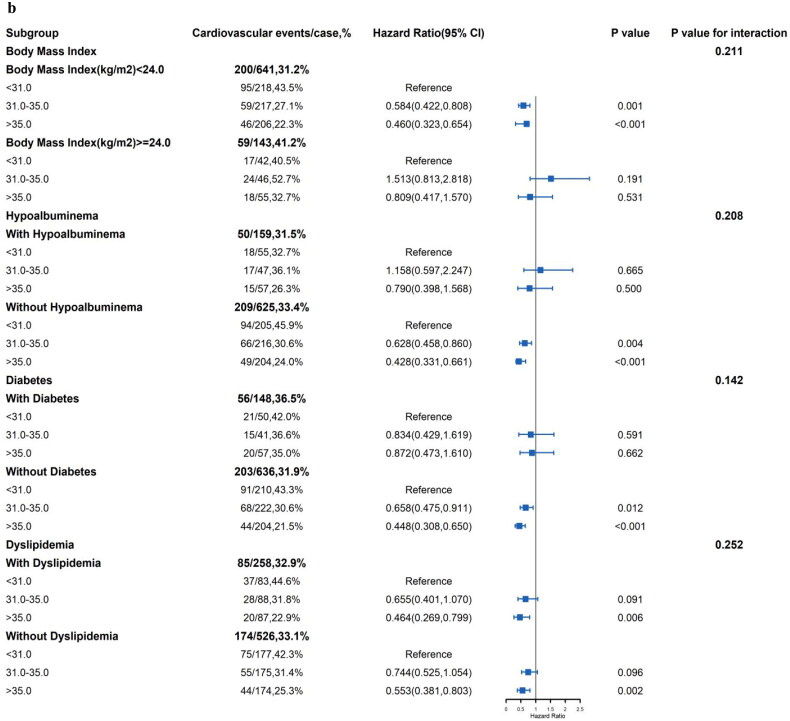

**Figure 4.**
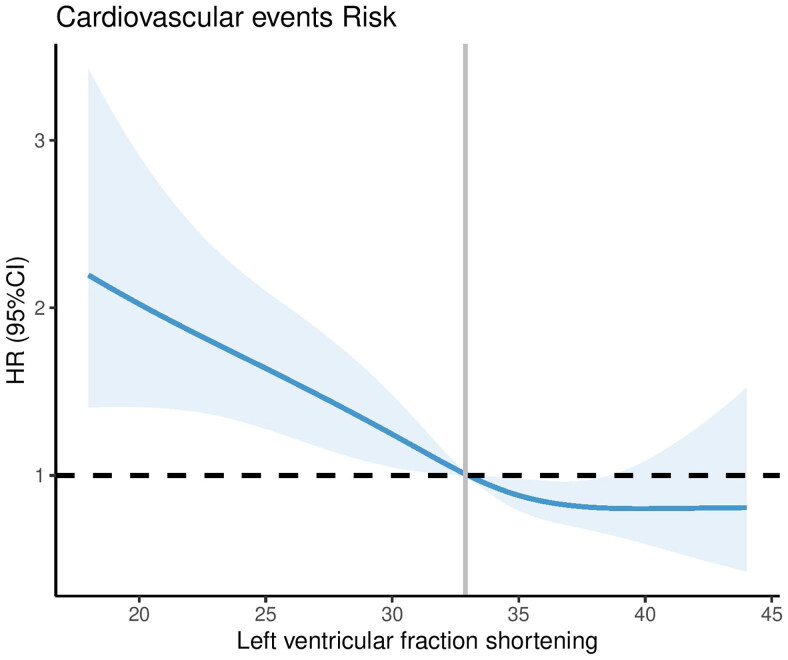
Association between the left ventricular fraction shortening with cardiovascular events risk in peritoneal dialysis patients. Hazard ratios are indicated by solid lines and 95% CIs by shaded areas.

We used the RCS model with four knots to simulate the relationship between the LVFS and the risk for CVEs. After adjusting for age, gender, body mass index, hypertension, diabetes mellitus, dialysis vintage, total cholesterol, low-density lipoprotein cholesterol, albumin, C-reaction protein, calcium, phosphorus, use of antihypertensive drugs and cholecalciferol drugs. The RCS model showed a non-linear relationship between LVFS and CVEs in PD patients, (Figure 4, nonlinear test, χ^2^ = 24.56, *p* non − linearity < 0.001). It can be observed that LVFS as a protective factor when LVFS values were > 32.89%, the risk of CVEs decreased significantly with the increase of LVFS values. At the same time, interestingly, LVFS as a risk factor when LVFS values were < 32.89%, the risk of CVEs increased significantly with the decrease of LVFS values.

The ROC curves were used to compare the predictive power of LVFS, EF, IVS, LVPW, LAD and RA. Compared with IVS, LVPW, LAD, and RA, the LVFS showed a better predictive power for predicting CV events. The optimal cutoff value of LVFS was 33.5% for predicting CV events, with a sensitivity of 52.2% and specificity of 64.5% (*p* < 0.001). In addition, EF, IVS, LVPW, LAD and RA in terms of predicting CV events are given in Supplemental Table 1.

## Discussion

In this cohort study of 784 PD patients with a median follow-up of 42.3 months, we demonstrated that decreased LVFS levels were significantly associated with higher incidence of CVEs in PD patients, especially among female, aged < 45 years, with hypertension and with dyslipidemia.

Studies have shown that dialysis patients have higher rates of arteriosclerotic outcomes and CVEs mortality [13]. A large number of cohort studies have showed that the incidence rate of CVEs in patients on PD is as high as 15.7–37.3% [14–16]. In this study, the prevalence of CVEs in PD patients was 33.0%, which was similar to the previous studies. To the best of our knowledge, the association between the LVFS and CVEs in PD patients was revealed for the first time in our study.

Cardiac structural and functional abnormalities is a well-recognized risk factor for mortality and adverse CVEs. Several previous studies have indicated that left atrial and ventricle enlargement can be used as a predictor of atrial fibrillation, hypertension, stroke, congestive heart failure, and cardiovascular death, which could be useful in clinical applications. In adidtion, left ventricular hypertrophy is a risk factor for cardiac functional abnormalities [11,17,18]. Previous studies have found that the echocardiographic parameter, LAD, has a good prognostic value for poor renal outcomes in CKD stage 3–5 patients [11]. In our study, LVFS was negatively correlated with LAD, the Cox regression model and ROC curve further indicated that LVFS had a better correlation with the occurrence of CVEs in PD patients. Zahra et al, [18] found that left atrial structure and function measures demonstrate significant associations with incident cardiovascular outcomes, independent of left ventricular metrics. In addition, in our study, even in the multivariate competing risk regression analysis for CVEs, the LVFS still showed better correlations with CVEs in PD patients. Furthermore, our study found that LVFS has a better correlation than other cardiac ultrasound indicators in peritoneal dialysis patients. Meanwhile, our research found that the LVFS as a protective factor when LVFS values were > 32.89%, it as a risk factor when LVFS values were < 32.89%, however, it provides some reference value for clinical evaluation of cardiac structure and function and risk of CVEs in PD patients. Our findings had extended the results of previous studies.

The potential mechanisms of association between LVFS and CVEs in PD patients in this study may be as follows. PD patients often have excessive volume load, whose poor prognosis is mainly due to its association with cardiovascular effects such as left ventricular hypertrophy, left ventricular systolic and diastolic dysfunction, pulmonary hypertension, and increased aortic stiffness [19]. In the meantime, some studies have suggested that overhydration may be one of the most important cardiovascular risk factors specifically in PD patients[4]. In our study, we speculate that LVFS, as one of the noninvasive echocardiographic parameters, cardiac function is better than LAD in evaluating CVEs in PD patients, and can be used to quickly analyze heart function using a straightforward method. In addition, ultrafiltration failure occurs in about one-third of patients with PD [20], and it may easily lead to hypertension and overhydration. This study found that compared with the other two groups, the patients in tertile1 had a higher prevalence of hypertension, suggesting that overhydration is related to CVEs in PD patients. Furthermore, we found a significant interaction between the LVFS and hypertension, and a significant detrimental effect of a low LVFS on CVEs was observed only in patients with hypertension on PD. However, increased cardiac output due to both anemia and hyperparathyroidism was found to cause ventricular dysfunction and increased ventricular wall thickness [21,22], ultimately leading to an increased risk of cardiovascular events. The low hemoglobin concentrations have been associated with an increased risk of major cardiovascular events [23]. The anemic state may lead to ventricular remodeling and cardiac insufficiency. Chronic anemia with hemoglobin < 10 g/dL is known to cause increased cardiac output, which leads to left ventricular hypertrophy [24]. Indeed, intact parathyroid hormone (iPTH) has a direct trophic effect on cardiomyocytes and fibroblasts, leading to intramyocardial arterial wall thickening, ventricular hypertrophy, and myocardial fibrosis. This study found that the patients in tertile1 had lower hemoglobin concentration and higher iPTH, is consistent with previous studies. Moreover, our previous published research articles have shown that the data from our center have certain significance in evaluating and predicting risk factors related to technical failure and mortality in peritoneal dialysis patients [25].

Continuous activation of inflammatory response is an important independent risk factor for cardiovascular abnormalities in dialysis patients, which contributes to excessive CVEs mortality in PD patients [4,26]. Volume overload, a common complication in PD patients, is itself associated with immune activation, leading to increased production of proinflammatory cytokines [27]. In a large cohort study of PD patients, inflammation determined by level of C-reactive protein and left ventricular hypertrophy synergistically increased the risk of all-cause mortality and cardiovascular death [27]. In addition, intraperitoneal perfusion of dialysate not only increases intra-abdominal pressure, but also increases systemic blood pressure due to increased total peripheral resistance. A large body of evidence indicates that patients with hypertension are characterized by endothelial dysfunction mediated by an impaired nitric oxide availability secondary to oxidative stress production, and dysfunction endothelium is an early marker of the development of atherosclerotic changes and can also contribute to CVEs [28]. However, inflammation, dyslipidemia, and atherosclerosis are closely related to cardiovascular disease [29]. Dyslipidemia is a major risk factor for the development of atherosclerotic disease [30], in our study, further in the subgroup analysis according to lipid metabolism disorder, the higher risk of CVEs with low LVFS was observed in PD patients with lipid metabolism disorder. Therefore, in the present study, LVFS, while assessing cardiac function, may also independently or integrally represent the prognostic value of all these factors in PD patients.

Our study has several strengths. First, this study is a single-center observational cohort study and a relatively large cohort of incident PD patients, which provided longitudinal study-based support. Second, our participants were a reliable group of patients undergoing PD coming from the real world, who had electronic health records from long-run medical centers. Our interview took place in these medical centers, which guaranteed reliability and were more representative of the real world. Third, our study observed robust finding that LVFS is independently associated with the risk of CVEs in peritoneal dialysis patients, especially those with woman, age <45 years, history of hypertension and the presence of dyslipidemia.

There were several limitations that require consideration. First, this was a single-center observational study, and the existence of center-specific effects cannot be completely excluded, and there are still some potential unknown or unmeasured confounding factors related to LVFS. Second, we collected baseline LVFS only and did not consider the effects of temporal changes in LVFS during follow-up. Third, the observed measures were limited, because brain natriuretic peptide, measures of cardiothoracic ratio, and types of cardiovascular events were excluded from the study. Fourthly, lack of therapeutic effect and could not evaluate the efficacy of medical treatment of patients. Last but not least, racial differences may have a certain impact on the occurrence of adverse events and the accuracy of prognosis evaluation.

In conclusion, our results found significant associations between the LVFS and CVEs in patients on PD, especially those with woman, age <45 years, history of hypertension and the presence of dyslipidemia. And the LVFS independently, or in combination with hypertension, has significant implications in predicting CVEs in PD patients.

## Supplementary Material

Supplemental Material

Supplemental Material
