# Supplementary material for: The association of left ventricular fraction shortening with cardiovascular events in peritoneal dialysis patients
Source: Ren Fail. 2023 Oct 1;45(2):2261786. doi: 10.1080/0886022X.2023.2261786 (PMC11001333; doi:10.1080/0886022X.2023.2261786)
Supplement: Supplemental Material [file IRNF_A_2261786_SM1934.pdf]

Supple table2 Cox proportional hazards models of baseline echocardiogram variables and cardiovascular events

Variables	Unadjusted Model		Model1		Model2		Model3	
	HR (95%CI)	*P*-value	HR (95%CI)	*P*-value	HR (95%CI)	*P*-value	HR (95%CI)	*P*-value
Continuous variable								
EF	0.970(0.957-0.983)	<0.001	0.968(0.955-0.981)	<0.001	0.968(0.954-0.982)	<0.001	0.969(0.955-0.981)	<0.001
Categorical variable								
EF tertile 1	Reference	-	Reference	-	Reference	-	Reference	-
EF tertile 2	0.732(0.549-0.975)	0.033	0.708(0.526-0.953)	0.043	0.712(0.529-0.959)	0.025	0.719(0.531-0.974)	0.033
EF tertile 3	0.601(0.442-0.816)	<0.001	0.575(0.420-0.787)	0.001	0.578(0.422-0.791)	0.001	0.594(0.433-0.817)	0.001
Continuous variable								
LAD	1.039(1.018-1.060)	<0.001	1.032(1.009-1.055)	0.006	1.036(1.014-1.058)	0.001	1.030(1.007-1.053)	0.009
Categorical variable								
LAD tertile 1	Reference	-	Reference	-	Reference	-	Reference	-
LAD tertile 2	1.223(0.888-1.685)	0.018	1.212(0.871-1.686)	0.255	-	-	-	-
LAD tertile 3	1.763(1.299-2.393)	<0.001	1.721(1.247-2.375)	0.003	1.714(1.109-2.196)	0.001	1.566(1.126-2.179)	0.008
Continuous variable								
LVD	1.049(1.029-1.069)	<0.001	1.047(1.025-1.069)	<0.001	1.049(1.028-1.070)	<0.001	1.025(0.998-1.053)	0.069
Categorical variable								
LVD tertile 1	Reference	-	Reference	-	Reference	-	Reference	-
LVD tertile 2	1.382(1.000-1.910)	0.050	-	-	-	-	-	-
LVD tertile 3	1.966(1.443-2.678)	<0.001	1.943(1.400-2.697)	<0.001	1.966(1.415-2.731)	0.001	1.795(1.283-2.511)	0.001
Continuous variable								
IVS	1.100(1.036-1.169)	0.002	1.096(1.024-1.172)	0.008	1.104(1.035-1.179)	0.003	1.091(1.021-1.167)	0.010
Categorical variable								
IVS tertile 1	Reference	-	Reference	-	Reference	-	Reference	-
IVS tertile 2	1.251(0.911-1.717)	0.167	-	-	-	-	-	-
IVS tertile 3	1.566(1.165-2.106)	0.003	1.633(1.189-2.243)	0.008	1.647(1.196-2.266)	0.002	1.589(1.148-2.199)	0.005
Continuous variable								
LVPW	1.096(1.036-1.169)	0.005	1.083(1.009-1.161)	0.027	1.095(1.023-1.172)	0.009	1.081(1.009-1.159)	0.027
Categorical variable								
LVPW tertile 1	Reference	-	Reference	-	Reference	-	Reference	-
LVPW tertile 2	1.049(0.764-1.442)	0.766	-	-	-	-	-	-
LVPW tertile 3	1.405(1.058-1.866)	0.019	1.405(1.041-1.896)	0.024	1.410(1.042-1.907)	0.026	1.371(1.008-1.865)	0.026

Note: Model 1 is adjusted for age, gender, body mass index, hypertension, diabetes mellitus; Model 2 is adjusted for age, gender, body mass index, hypertension, diabetes mellitus, dialysis vintage, total cholesterol, low-density lipoprotein cholesterol, albumin; Model 3 is adjusted for age, gender, body mass index, hypertension, diabetes mellitus, dialysis vintage, total cholesterol, low-density lipoprotein cholesterol, albumin, C-reaction protein, calcium, phosphorus, use of antihypertensive drugs and cholecalciferol drugs. Abbreviations: CI: confidence interval; HR: hazard ratio; EF, ejection fraction; LAD, left atrial diameter; LVD, left ventricular diameter; IVS, interventricular septum thickness; LVPW, posterior wall thickness of left ventricle. $P < 0.05$ was considered statistically significant.

Information Classification: General
