# Supplementary material for: The association of left ventricular fraction shortening with cardiovascular events in peritoneal dialysis patients
Source: Ren Fail. 2023 Oct 1;45(2):2261786. doi: 10.1080/0886022X.2023.2261786 (PMC11001333; doi:10.1080/0886022X.2023.2261786)
Supplement: Supplemental Material [file IRNF_A_2261786_SM1920.pdf]

Supple table1 AUC using ROC curve analyses to predict cardiovascular events

Variables	cardiovascular events			
	AUCs	SE	95%CI	*P*-value
LVFS	0.596	0.022	0.554-0.639	<0.001
LAD	0.587	0.022	0.544-0.629	<0.001
Ejection fraction	0.592	0.022	0.549-0.635	<0.001
Interventricular septum thickness	0.569	0.022	0.526-0.612	<0.001
Posterior wall thickness of left ventricle	0.565	0.022	0.521-0.608	0.003
Inner diameter of right atrium	0.550	0.022	0.506-0.593	0.024

Abbreviations: AUC, Area under the curve; ROC, receiver operator characteristic curve; SE, Standard error; CI: confidence interval

Information Classification: General
